# *FGD1* Variant Associated With Aarskog–Scott Syndrome

**DOI:** 10.3389/fped.2022.888923

**Published:** 2022-07-14

**Authors:** Yilin Zhu, Qingqing Chen, Haiyan Lin, Huifei Lu, Yangbin Qu, Qingfeng Yan, Chunlin Wang

**Affiliations:** ^1^Department of Pediatrics, The First Affiliated Hospital, College of Medicine, Zhejiang University, Hangzhou, China; ^2^Department of Pediatrics, The First People's Hospital of Wenling, Taizhou, China; ^3^College of Life Sciences, Zhejiang University, Hangzhou, China; ^4^Key Laboratory for Cell and Gene Engineering of Zhejiang Province, Hangzhou, China

**Keywords:** *FGD1*, short stature, Aarskog-Scott syndrome, CDC42, JNK1

## Abstract

**Background:**

Aarskog–Scott syndrome, a rare X-linked genetic disorder, is identified by combined clinical manifestations of short stature, facial, skeletal, and genital anomalies. Annually, two or three new cases are diagnosed with Aarskog–Scott syndrome, which is associated with *FGD1* variants. However, there is no specific treatment for Aarskog–Scott syndrome due to its unclear mechanism.

**Methods:**

Clinical data were collected when the patient first visited the hospital. Trio whole-exome sequencing and Sanger sequencing were performed for the genetic cause of disease. To evaluate the pathogenicity of the variants *in vitro*, stable cell lines were constructed using lentivirus infection in 143B cell. Furthermore, Western blot was used to verify the expression of signaling pathway-related proteins, and the transcription levels of osteogenic-related genes were verified by luciferase reporter gene assay.

**Results:**

A 7-year-old boy was manifested with facial abnormalities, intellectual disability, and short stature (−3.98 SDS) while the growth hormone level of stimulation test was normal. Trio whole-exome sequencing and Sanger sequencing identified a variant (c.1270A>G, p.Asn424Asp) in *FGD1* gene. The Asn424 residue was highly conserved and the hydrogen bond in the FGD1 variant protein has changed, which led to decrease in the interaction with CDC42 protein. *In vitro* study showed that the Asn424Asp variant significantly decreased the transcription levels of *OCN, COL1A1*, and ALP *activity*, and it activated the phosphorylation of JNK1.

**Conclusion:**

Molecular biological mechanisms between abnormal expression of *FGD1*and Aarskog–Scott syndrome remain poorly understood. In our study, c.1270A>G variant of *FGD1* resulted in Aarskog–Scott syndrome, and the analysis of pathogenicity supports the deleterious effect of the variant. Furthermore, we demonstrated the weakened affinity of the mutant FGD1 and CDC42. Decreased expression of osteogenic-related gene and abnormal activation of JNK1 were also shown in this work.

## Introduction

Aarskog–Scott syndrome (AAS, OMIM 305400), also termed as faciogenital dysplasia, is a male-predominant X-linked developmental disorder. It is rare, having a population prevalence of 1/25,000 or less (1). Children with AAS have heterogeneous clinical manifestations. Short stature, craniofacial, genital, and skeletal anomalies are the classically characteristic combination (2). With respect to short stature, the most common symptom in the disease; it is disproportionate, with an increased upper-to-lower segment ratio and shortened distal extremities (3, 4). Abnormalities emerge when children with this condition are 2–4 years old, with a delayed peak of pubertal growth spurt (5). There are individual differences in the growth hormone (GH) treatment for AAS due to the normal level of GH. However, previous studies have shown that GH therapy can promote growth in children with AAS (3). Therefore, the study of pathogenic mechanisms is desirable and would be expected to help in the formulation of more effective treatment regimes. So far, only one causal gene, *FGD1*, has been identified, but the relationship between aberrant expression of *FGD1* and AAS remains unclear.

The *FGD1* gene (faciogenital dysplasia 1, OMIM 300546), located at Xp11.21, encodes the RhoGEF and PH domain-containing protein 1 (6). The FGD1 protein is made up of a proline-rich region, Dbl homology (DH), and pleckstrin homology (PH) domains, a FYVE-finger domain, and a second PH domain (PH2) from the N-terminus to the C-terminus (7). As a member of the guanine nucleotide exchange factor family, the FGD1 protein specifically binds to cell division cycle 42 (CDC42), a member of the Ras homology (Rho) family of GTPase protein (8, 9). The binding of CDC42 to the DH domain of FGD1 catalyzes the exchange from GDP-bound (inactive) to GTP-bound (active) forms, which leads to the activation of the downstream signal pathway (10, 11). FGD1/CDC42 has been reported to be involved in numerous signaling pathways, including the actin cytoskeleton organization, cell polarization, vesicular trafficking, cell cycle progression, and gene expression (12–14). Furthermore, current research suggests that FGD1 may be a critical regulator of events modulating extracellular matrix, which is already known as an important factor in skeletal formation (15, 16).

In this report, detailed clinical and molecular genetic analysis using whole-exome sequencing (WES) technology was performed on a Chinese child with AAS. Gene sequencing revealed a variant in the *FGD1* gene c.1270A>G (p. Asn424Asp). Coincidentally, a boy, also diagnosed with AAS, has the same mutation site and has been previously reported in a case report (17). To verify the pathogenicity of the variant, we performed a series of experiments *in vitro*.

## Materials and Methods

### Subjects

The study was authorized by the Ethics Committee of The First Affiliated Hospital, Zhejiang University, China, which followed the Declaration of Helsinki principles. Written and informed consent was obtained from the proband's parents for publication of this case.

### Clinical Evaluation

A detailed clinical evaluation was collected at the time of diagnosis, including medical history and clinical presentations. Physical examination, laboratory and radiological imaging tests were recorded, as well as magnetic resonance imaging (MRI).

### Cytogenetic and Molecular Studies

Whole-exome sequencing of a proband–parent trio was used to search for the causative gene. The genomic DNA of the proband and his family members was isolated from peripheral blood samples using a TaKaRa blood genome DNA extraction kit (TaKaRa, USA). A total amount of 3 μg DNA was used for exome capture with the SureSelect Human All Exon V6 kit (Agilent Technologies), and then the prepared target libraries were sequenced on Illumina HiSeq X Ten (Illumina, San Diego, CA, USA) at an average depth of 100× , according to the instructions of the manufacturer. Whereafter, the results of Sanger sequencing were analyzed using Chromas Lite v2.01 (Technelysium Pty Ltd, Tewantin, QLD, Australia). The description of the Sanger sequencing variant detected in the *FGD1* gene was made on the basis of the NCBI entry NG_008054.1 (NM_004463.3). The description of the variant was conducted according to the Human Genome Variation Society sequence variant nomenclature (18).

### Conservative and Pathogenicity Analysis of the Variant

The protein sequences of FGD1 in different vertebrate species were downloaded from the UniProt Knowledge Database (https://www.uniprot.org/). A multiple-sequence alignment was created using the ClustalX program (ftp://ftp-igbmc.u-strasbg.fr/pub/ClustalX/). Results from Sequence alignment were displayed online using ConSurf (http://consurf.tau.ac.il/) (19). The variant pathogenicity predictors were carried out by the webserver PREDICT-SNP (https://loschmidt.chemi.muni.cz/predictsnp1/) (20).

### Variant Classification

The pathogenicity of the variant was classified according to the classification of the latest version of ACMG (American College of Medical Genetics and Genomics) (21). The findings were divided into five categories, namely, (1) pathogenic, (2) likely pathogenic, (3) uncertain significance, (4) likely benign, and (5) benign.

### Homologous Modeling of Human FGD1

The 3D structure of the FGD1 protein globally was generated by Threading ASSEmbly Refinement (I-TASSER) (https://zhanglab.ccmb.med.umich.edu/I-TASSER/) (22) due to the lack of an existing experimental structure. The UniProt database was the source for attaining the amino acid sequence of wildtype human FGD1. Meanwhile, the local structural, stability, and flexibility analysis of mutant FGD1 was predicted by the online server Dynamut (http://biosig.unimelb.edu.au/dynamut/) (23). The structures of the protein were rendered in PyMOL 2.4 for analysis and illustration.

### Lentivirus Infection

The full-length cDNA fragment of human *FGD1*, excluding the stop codon, was subcloned into a pCDH vector for expression. A 3× FLAG tag was added to the C-terminal of pCDH-*FGD1*. An In-Fusion HD Cloning kit (TakaRa, USA) was used to construct the mutational pCDH-*FGD1*. Transfection was done using three plasmid systems, including packaging plasmid (psPAX2), envelope plasmid (pMDG2), and the pCDH plasmid containing *FGD1*-WT-cDNA or *FGD1*-mut-cDNA. The three plasmids were co-transfected at a ratio of 5:3:2 into HEK-293T cells, and the medium was collected after 48 h. Subsequently, the virus supernatant was added into 143B cells after being filtered. After 24 h, the stably transfected cells were selected with a suitable concentration of 6 μg/ml puromycin.

### Dual-Luciferase Reporter Gene Assays

Analogously, the plasmids of pGL4-OCN-Luc and pGL4-COL1A1-Luc were constructed by ligating promoter sequences of *OCN* and *COL1A1* into the pGL4.48 vector (Promega, USA). Prior to transient transfection, 143B cells were plated into 24-well-plates at a confluency of 70–80%. The reconstructed reporter plasmids and helper plasmid pGL4.74 [hRluc/TK] (Promega, USA) were co-transfected at a ratio of 50:1 (a total of 500 ng DNA) by using Lipofectamine™ 3,000 transfection reagent (Thermo Fisher, USA). Culture medium was replaced with fresh medium 8 h later. The Dual-Glo® Luciferase Assay System (Promega, Cat# 2920) was performed 48 h after transfection.

### Measurement of Protein Interaction in Cells

NanoLuc® Binary Technology (NanoBiT) (Promega, Cat# N2014), a novel technology that was used to analyze the interacted relation in proteins. It contains two complementary subunits, LgBiT (18 kDa) and SmBiT (3.6 kDa). *FGD1*-LgBiT-C (or *FGD1*-mut-LgBiT-C) and CDC42-SmBit-N were constructed, as well as the confluency of HEK-293T cells in 24-well reached at ~70–80% before transfection. The transfected method was the same as above, except the ratio of DNA was 1:1. Then, 48 h after transfection, the luciferin was measured by a Multimode Plate Reader VICTOR Nivo (ND-1000, Thermo Fisher) after the addition of the diluted substrate.

### Cell Culture

HEK-293T, 143B cell lines were cultured in 60 mm cell culture dish, which was added with 4 ml HG-DMEM (Gibco) supplemented with 10% fetal bovine serum. In addition, the CO_2_ cell culture incubator was set to 37°C and 5% CO_2_. The cells were passaged when cell confluence reached 90%.

### Western Blotting

A total protein Extraction Kit (Abcam) was used to extract the proteins from cells according to instructions, and protein quantification was conducted by bicinchoninic acid assay. The supernatant was resolved on 10% SDS-PAGE and transferred from the gel to polyvinylidene difluoride later. The membranes were then incubated in TBS-5% skim milk for 1 h at room temperature. The first antibodies were incubated overnight at 4°C, and the secondary antibody was incubated for 2 h at room temperature. The primary antibodies included p-JNK1 (1:1,000, CST), GADPH (1:2,000, ABclonal), FGD1 (1:2,000, ABclonal), and T-JNK1 (1:1000, CST). The second antibodies, goat anti-mouse/rabbit IgG, were purchased from Abcam.

### Alkaline Phosphatase (ALP) Activity Assay

The ALP activity of stable transfected cell lines which was cultured with osteogenic medium (Stemcell, Canada) was tested by an ALP activity assay (Beyotime, Shanghai, China). Cells in 24-well-plates were collected and no protease inhibitor cocktail lysis buffer was used for lysis. Then, 50 μl/well-Supernatant protein was fully mixed in the 96-well-plate with the substrate of 50 μl/well of *p*-nitrophenol. After incubation at 37°C for 30 min and adding 100 μl of stop solution, the absorbance of the mixture was detected at 405 nm (BioTek, USA).

### Statistical Analysis

All the statistical analyses were performed by *t*-test (two-tailed, unpaired) in the GraphPad Prism 8 program. ImageJ was used to handle and process the Western blotting images. All the experiments were repeated at least three times independently. It was considered significant that the *p*-value was lower than 0.05 (^*^p < 0.05, ^**^p < 0.01, ^***^p < 0.001).

## Results

### Clinical Evaluation

**Figure 1:**
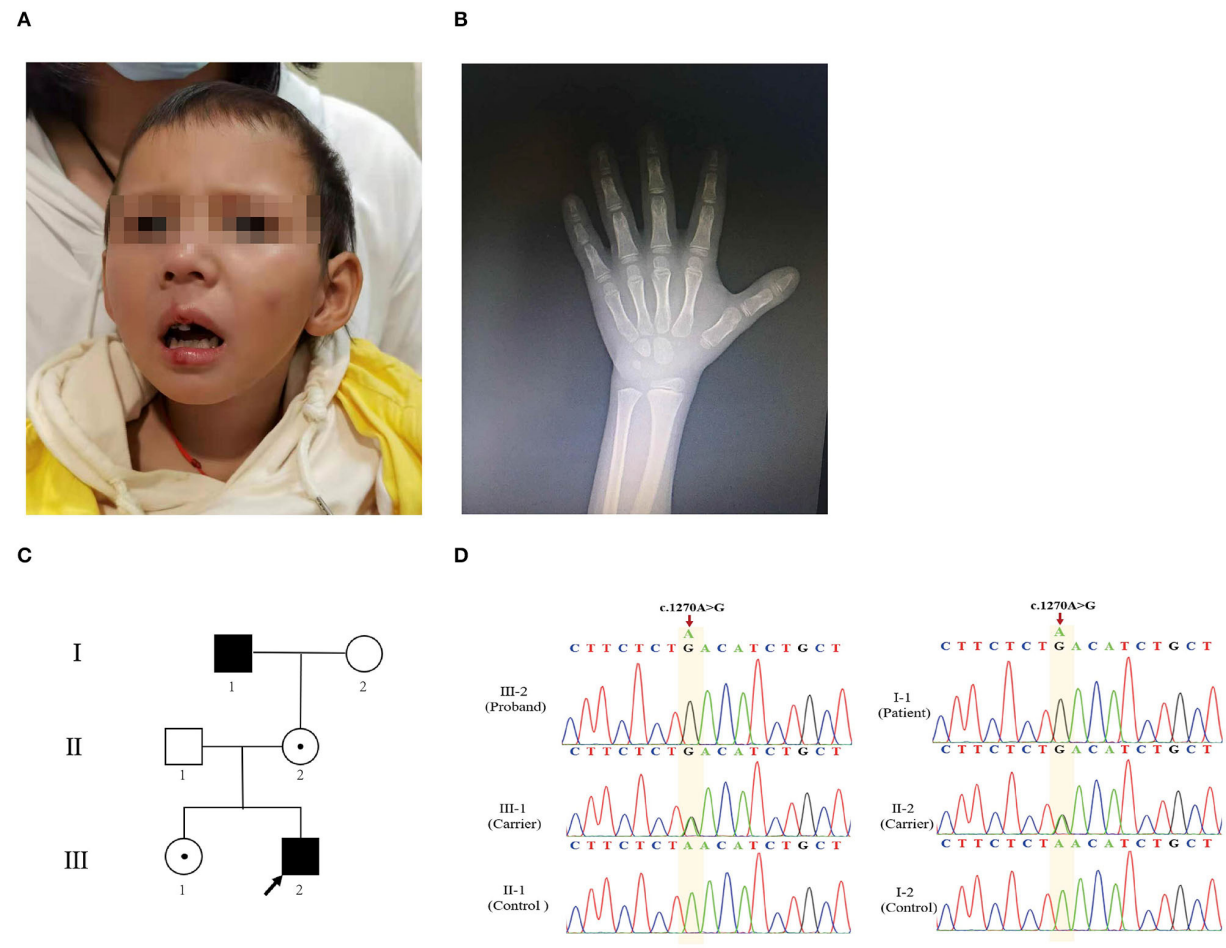
Clinical characteristics of the proband. **(A)** Image of the proband. Facial abnormalities included triangular face, long philtrum, low-set ears, and short nose. **(B)** Hand X-ray of the patient. **(C)** The pedigree analysis and pedigree symbols. The squares and circles refer to males and females, respectively, and the arrow indicates the proband. A filled symbol represents a person affected with AAS, and the center of the black spot circle indicates carrier status. **(D)** Sanger sequencing chromatogram demonstrates mutation from affected individuals and heterozygous variant from unaffected family members.

The proband, a 7-year-old boy of healthy non-consanguineous parents, was referred to our hospital complaining of growth retardation for 6 years. He was born at full term by spontaneous vaginal delivery with a birth weight of 3.75 kg. His weight was 17.8 kg (−2.0 SDS) and height 105.7 cm (−3.98 SDS). The proband had short stature (HP:0004322), facial abnormalities, including triangular face (HP:0000325), long philtrum (HP:0000343), low-set ears (HP:0000369), and short nose (HP:0003196) (Figure 1A). Wechsler Intelligence Scale for Children Test revealed intellectual disability (HP:0001249) (IQ = 70). The peak-stimulated GH level of the stimulation tests was 13.57 ng/ml, and the bone age was about 3 years and 8 months (Greulich–Pyle method) (Figure 1B). Additionally, there were no abnormalities in the functions of the thyroid, liver, gonad, adrenal gland, blood glucose, or electrolytes. Further imaging examination (pituitary MRI and spinal X-ray) showed nothing unremarkable. Notably, his family members were all short: his father's height was 160 cm (−2.08 SDS), his mother's height was 150 cm (−1.96 SDS), his elder adult sister's height was 153 cm (−1.41 SDS), and his maternal grandfather's height was 150 cm (−3.72 SDS). However, they were physically healthy without intellectual disability and other malformations.

### A Missense Mutation in *FGD1* Was Identified as the Causal Variant

The proband (III-2) (Figure 1C) had severe short stature, intellectual disability, and facial abnormalities, all of which suggested a genetic factor and necessitated genetic testing. To determine the proband's genetic etiology, trio-WES and Sanger sequencing were performed on the proband and his parents. Peripheral blood samples were collected and genomic DNA was extracted subsequently. The WES analysis of the patient revealed 35,224 variants in the exome region. Among these, a total of 33,250 (94.39%) single-nucleotide polymorphism (SNP) loci and 1,974 indel variations were identified. Next, 24,356 SNPs and 1,010 InDels were screened out, which were potentially affecting coding sequences (i.e., non-synonymous, nonsense, or located in the canonical splice-site region). A total of 162 SNPs and 8 InDels were deleterious mutations according to the PROVEAN, SIFT, PolyPhen-2, MutationTaster, and protein structure prediction software. We then screened 22 SNPs and 3 InDels based on genetic patterns. Then, 11 corresponding SNPs were filtered out based on the minor allele frequency of SNPs when <0.01 was used to exclude common mutations based on the 1000G_ALL database (http://www.internationalgenome.org/) (Supplementary Table S1). Based on the clinical manifestation and heredity pattern, *FGD1* (chrX: 54,494,287 in GRCh37, c.1270A>G, p.Asn424Asp) was considered as the most relevant candidate gene for the proband (Supplementary Figure S1A). Because of the X-linked recessive inheritance pattern associated with the *FGD1* gene and the proband's maternal grandfather (I-1) having severe short stature, Sanger sequencing was used to confirm whether the proband's other family members carried the same mutation. The results indicated that the proband's variant came from his maternal grandfather (I-1). Both of them were hemizygous males. His mother (II-2) and sister (III-1) were both carriers (Figure 1D). This variant was classified as likely pathogenic according to the variant guidelines of the ACMG (PM1, PM2, PP1, PP3). Additionally, the mutation was predicted to be pathogenic by PredictSNP (score 0.65), PolyPhen-1 (score 0.67), PhD-SNP (score 0.86), SIFT (score 0.53), and SNAP (score 0.72) (Supplementary Figure S1B).

### Sequence Conservation and Three-Dimensional Structure of FGD1

**Figure 2:**
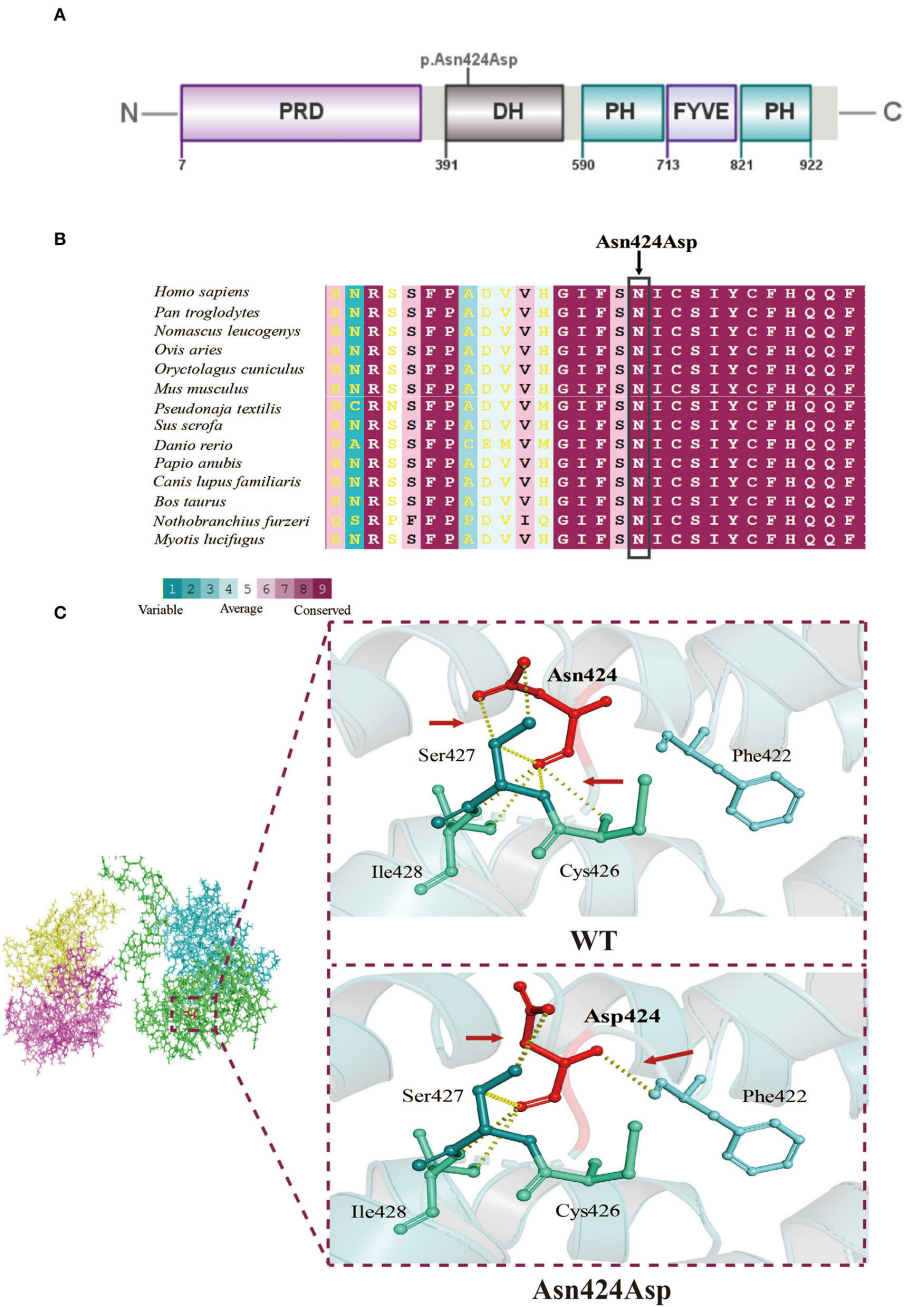
Molecular analysis. **(A)** A schematic diagram showing the location of the mutation site. **(B)** Interspecific conservation analysis of the p.Asn424Asp mutation in FGD1, highlighted by the box. **(C)** Three-dimensional structure model of the local structural changes between wildtype and mutant FGD1 protein, as predicted by Dynamut. Red arrows represent the difference in hydrogen between the wildtype and mutant.

The mutation site of the FGD1 protein (p. Asn424Asp) was located in the DH domain (Figure 2A). To identify the importance of the position in the domain, we compared FGD1 protein sequences in 58 vertebrates. Figure 2B shows a comparison of FGD1 protein sequences in representative species, including *Homo sapiens, Pan troglodytes, Ovis aries, Oryctolagus cuniculus, Mus musculus, Pseudonaja textilis, Sus scrofa, Danio rerio, Papio anubis, Canis lupus familiaris, Bos taurus, Nothobranchius furzeri*, and *Myotis lucifugus*. The conservation of the variant position in FGD1 was conservatively scored using ConSurf, which indicated that the residues at site Asn424 of FGD1 were highly conserved through the represented vertebrates.

The biological function of a protein is determined by the secondary structure of the protein. Three-dimensional structural modeling of both normal and mutant FGD1 proteins in order to exhibit the consequences of the N424D mutation on the tertiary or quaternary structure of the FGD1 protein was done (Figure 2C). Typically, the oxygen atom and nitrogen atom of Asn (p.424) are held together with the nitrogen atom of Cys (p.426) and the carbon atom of Ser (p.427) by hydrogen bonds. The p.Asn424Asp variant would not only disrupt the hydrogen bonds but also build a new association with Phe (p.422), which resulted in structural changes inevitably.

### FGD1^N424D^ Variant Upregulates p-JNK1

**Figure 3:**
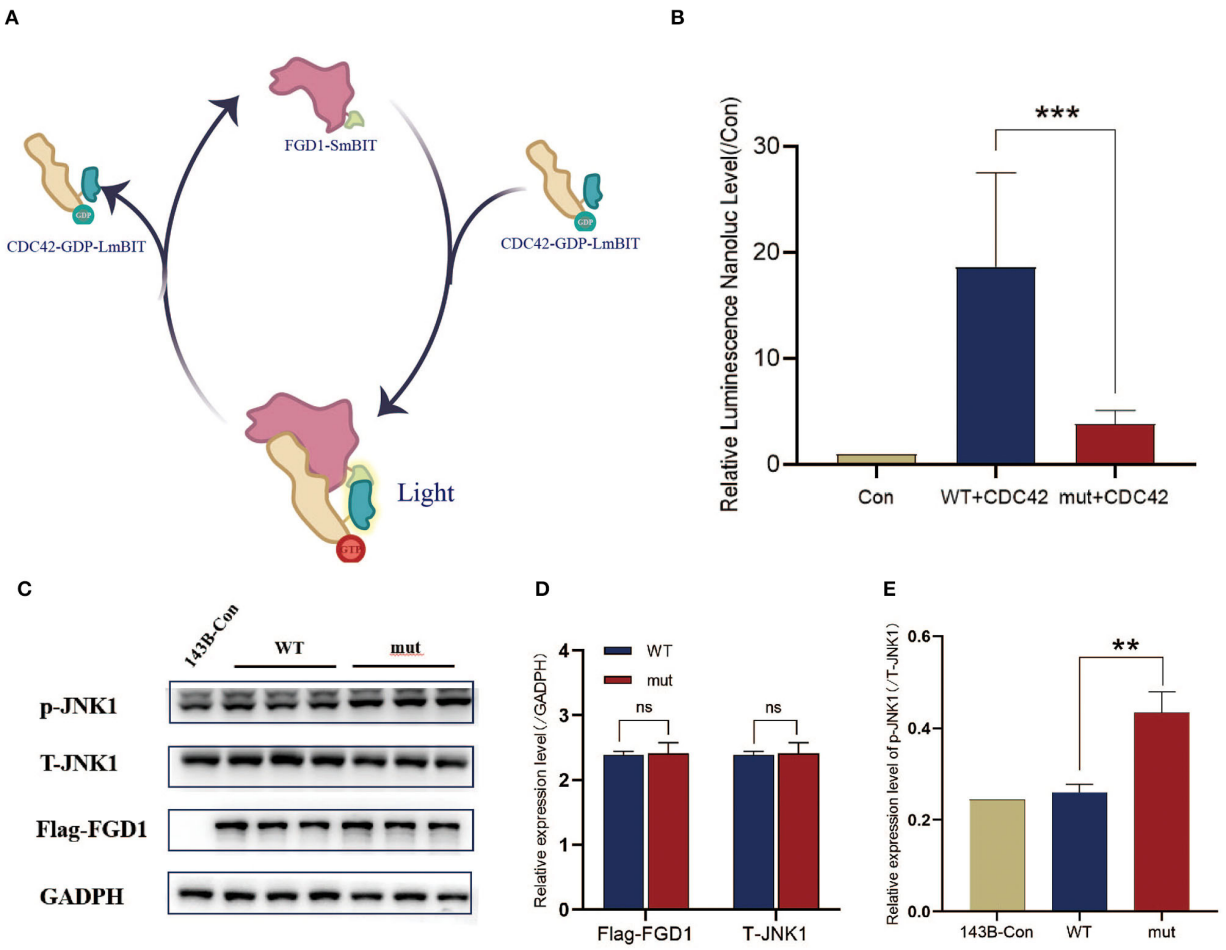
The interaction of the variant of FGD1 and CDC42 has been decreased, which might be contributing to the increase of p-JNK1. **(A)** Schematic diagram of interaction between FGD1 protein and CDC42 protein by NanoLuc® Binary Technology. **(B)** To facilitate the comparison, luciferase values were normalized to Control values (100%), histogram and bar chart represent the relative value of FGD1-CDC42 binding capacity. **(C)** Western blotting analysis of Flag-FGD1, p-JNK1, and T-JNK1 in 143B cell lines. **(D,E)** Quantified analysis of Western blotting of Flag-FGD1, p-JNK1, and T-JNK1 in 143B cell lines. **p < 0.01, and ***p < 0.001.

It is generally accepted that FGD1 is a highly specific CDC42 guanine exchange factor, and the binding domain is located in DH, similar to the mutational domain (24). To clarify the effect of the variant on combinative activity, we examined the mutant FGD1 protein-binding ability of CDC42 according to the schematic diagram (Figure 3A). This finding indicated that mutations in the *FGD1* gene led to a significant decrease in the binding capacity of CDC42 (Figure 3B). Activated-CDC42 activates multiple downstream signaling pathways by interacting with effector proteins. JNK1, which is downstream of CDC42, is known to be associated with bone development (25). To further determine the downstream pathways, the expression level of JNK1 and phosphorylated JNK1(The 183/Tyr 185) was measured by Western blotting. The results showed a significantly higher level of phosphor-JNK1 in the FGD1 mutant group (1.109 ± 0.0614 vs. 0.8800 ± 0.02828, *t* = 11.93, *p* < 0.01), while the total cellular level of JNK1 remained the same in the wildtype and mutant groups (Figures 3C–E).

### The *FGD1* Variant Inhibits the Activity of ALP and Downregulates *OCN* and *COL1A1*

**Figure 4:**
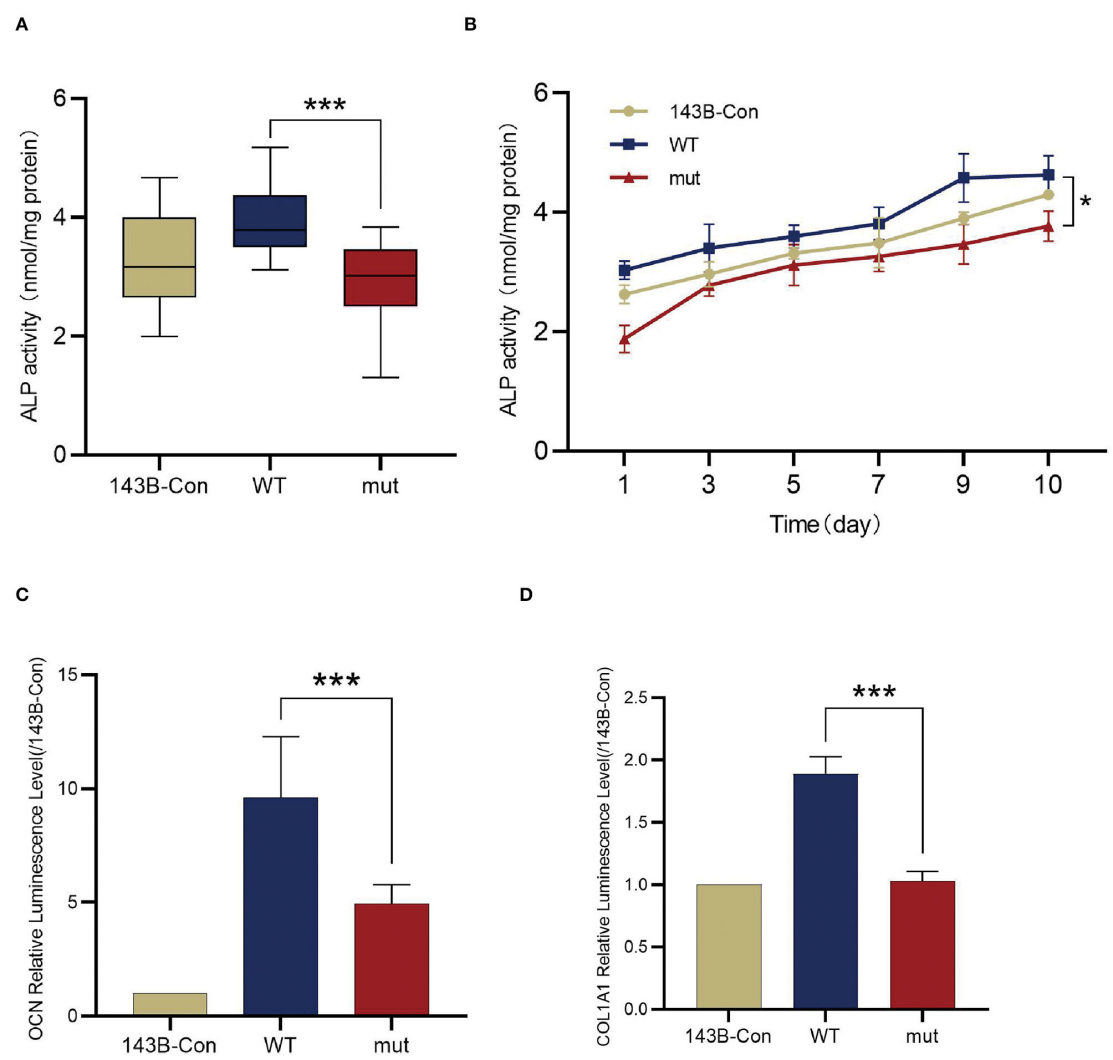
Comparison of the osteogenic differentiations between wildtype 143B and mutant 143B. **(A)** ALP activity determined by ALP activity assay. **(B)** ALP activity was measured every other day for 10 days since stable cell lines cultured with osteogenic medium. **(C)** The transcription of *OCN* was evaluated by dual luciferase reporter gene assay. **(D)** The transcription of *COL1A1* were evaluated by dual luciferase reporter gene assay. ***p < 0.001.

To determine the influence of the *FGD1* variant in skeletal development, FGD1^wt^ and FGD1^N424D^ overexpression was performed in a human osteosarcoma cell line, 143B. For the osteogenic makers, the transcriptional level of *OCN, COL1A1*, and ALP activity was detected (Figures 4A–D). Cell lysates were used for ALP activity measurement by an ALP activity assay kit. We examined the ALP activity in 143B-WT and 143B-mut, and the result demonstrated that the mutant has an obvious decrease in ALP activity compared with the wildtype (2.887 ± 0.7146 vs. 3.947 ± 0.5884, *t* = 6.564, *p* < 0.001) (Figure 4A). For dynamic changes of ALP, test data were collected for 10 consecutive days since the cell lines cultured with osteogenic medium. The results showed the ALP activity of the groups rose first, then gradually went steady, but the mutant group was below throughout (Figure 4B). In addition, the transcription level of *OCN* and *COL1A1* was analyzed by dual-luciferase reporter gene assays. *OCN* and *COL1A1* promoter luciferase plasmids were expressed in stable cell lines. Compared with the wildtype group in expression of *OCN*, the mutant group significantly decreased (4.937 ± 0.8401 vs. 9.616 ± 2.677, *t* = 4.124, *p* < 0.001). Corresponding to this, the level of *COL1A1* transcription was significantly lower in the *FGD1* mutation group compared with the wildtype group (1.027 ± 0.07944 vs. 1.892 ± 0.1351, *t* = 11.03, *p* < 0.001) (Figures 4C,D).

## Discussion

In this study, we described a 7-year-old boy with AAS in a Chinese family exhibiting severe short stature, facial abnormalities, and intellectual disability. WES and Sanger sequencing confirmed the disease-causing mutation in *FGD1* (c.1270A>G). As a consequence of a wide variety of clinical features, a clinical suspicion of AAS was reliant on fulfilling a few primary and secondary criteria according to Teebi presented in 1993 (26). The diversity of clinical manifestations means that AAS is still difficult to diagnose and requires differential diagnosis, such as Robinow syndrome, Noonan syndrome, and SHORT syndrome. Therefore, this highlights the importance of genetic testing. Among the appearance characteristics of the boy, short stature, short nose, and long philtrum matched primary criteria, low-set ears belonged to the additional, and only triangular face was slightly different from the common feature of round face (26–28). Compared with the typical clinical presentation of affected patients, the boy did not have genital hypoplasia (shawl scrotum) and skeletal anomalies (short/broad hands). Despite the boy meeting a few diagnostic criteria, AAS remained unclear. It can be hard to distinguish between other disorders according to these clinical manifestations alone (1). Based on the genetic testing report, clinical manifestations, and family history (maternal grandfather with severe short stature), the boy was diagnosed with AAS caused by the *FGD1* variant. Furthermore, stubby metacarpals, bilateral swollen testicles, and inguinal hernia were reported previously in a patient with AAS who carried the same variant, in addition to short stature and facial abnormalities (17). However, while the boy in our study had intellectual disability, the other patient who carried the same variant only had mildly delayed motor development (walking at the age of 15 months).

In the boy's family, his maternal grandfather with severe short stature was also diagnosed as AAS, carrying the same *FGD1* variant. His maternal grandfather did not have facial abnormalities and intellectual disability, which could be due to clinical heterogeneity. However, milder manifestations could also be related to the increasing age (29). Intriguingly, his mother and sister also carried the same *FGD1* variant, who only presented with mild short stature. This was also observed in other female carriers (2, 17, 30). Alternatively, clinical phenotypes of female patients with AAS, in general, seemed relatively mild, which may be associated with X chromosome inactivation (31, 32).

Associated intellectual disability appears to be rare. Only few independent studies have been reported. However, no direct evidence was given to the correlation between AAS and intellectual disability (33–36). Aside from the *FGD1* gene, 4 of the 11 corresponding SNPs sites were associated with intellectual disability (Supplementary Table S1). However, they were unable to explain all of the boy's clinical manifestations by analyzing clinical presentation and genetic pattern. For example, the *CCDC41* gene variants would be present in nephronophthisis (37); *LSS* gene variants can result in alopecia (38); *LTBP3* gene variants are characterized by hypoplastic amelogenesis imperfecta (39); *NTRK1* gene variants have been found to cause congenital insensitivity to pain (40).

In this study, consistent with the clinical phenotype, pathogenicity assay, structural modeling, and conservation analysis show the deleterious effects. Clinical and molecular evidence indicated the pathogenicity of the c.1270A>G variant in *FGD1*. However, how *FGD1* variants result in the phenotype of skeletal anomalies remains unclarified. We presented the evidence that the mutation in *FGD1* weakened the interaction with CDC42 and tend to impair catalytic role. Consistent with the previous study showing negative mutants of CDC42 inhibit CDC42 signaling and suppressed osteogenesis, our study manifests the attenuated CDC42 signaling in skeletal dysplasia (41). Although dysregulation of the FGD1/CDC42 signaling pathway is suspected to be associated with skeletal defects in AAS, whether the downstream signaling molecules and the mechanism of mutations in *FGD1* might influence bone development remain to be elucidated (42). It is well-known that CDC42 regulates MAPK signaling pathways, including P38, ERK, and JNK, that might have an important impact on numerous cellular activities. Studies on the function of P38 and ERK in bone development were popular compared to JNK. Nevertheless, increasing number of research suggests that JNKs are critical mediators of osteoblast activity (43, 44). In mammals, JNK1, JNK2, and JNK3 are three distinct genes that encode JNKs. Among them, JNK1 and JNK2 are distributed widely in most cell types, whereas JNK3 is limited to the brain and testes (45). In regulating cell activities in particular cell cycle and apoptosis, active-CDC42 positively regulates p-JNK while during some cell life activities, such as hepatocyte differentiation, the inhibition of CDC42 activity leads to upregulate p-JNK (46). Similarly, our study showed decreased affinity of FGD1 and CDC42 promoted JNK1 phosphorylation. Therefore, we deduce that the variant of *FGD1* influences bone development *via* the increase in phosphorylation-JNK1. However, whether it is a direct or an indirect effect awaits further experiments.

To further explore the downstream characterization of molecular changes that adversely affect the formation of multiple skeletal structures, the follow-up experiments were performed. RUNX2 is an essential transcription factor involved in mesenchymal progenitors toward the osteogenic lineage (47). No significant discrepancies of RUNX2 were detected in the literature, which demonstrated that p-JNK1 did not affect the expression of RUNX2, but further decreased its transcriptional activity (25). Therefore, some osteogenic markers, the target genes of *RUNX2*, have been detected in our study. ALP, an early marker for osteogenic differentiation, is widely recognized to represent the degree of osteogenic differentiation (48). As shown here, the mutation in *FGD1* led to decreased ALP activity compared to wildtype. Furthermore, osteogenic induction medium enhanced the ALP activity increase gradually to a stable level, but the activity of ALP in the mutant set is consistently lower than the others. The previous literature illustrated that the mutation in the *ALP* gene could result in skeletal disorder, including short stature (49). Besides, COL1A1 and OCN are largely considered as the maturation state markers or transcription factors of osteoblasts. Our cellular experiments confirmed that the variant identified in *FGD1* that contributed to the reduced transcription level of *COL1A1* and *OCN* plays the key role in skeletal development. Damian et al. reported that the mutations in *COL1A1* affect bone growth by bone deformities, and this was in line with the subsequent studies, which showed the evidence of a cumulative affected on short stature caused by mutations in *COL1A1* (50, 51). Similarly, downregulated *OCN* often occurs in the mechanism studies of idiopathic short stature (52–54).

## Conclusion

We reported a patient with AAS due to the c.1270A>G variant in *FGD1* gene predicted to be pathogenic by bioinformatics programs. Additionally, we found that the variant weakened the interaction with CDC42 and decreased the expression of osteogenic-related genes through abnormal activation of JNK1 *in vitro*.

## Data Availability Statement

The datasets for this article are not publicly available due to concerns regarding participant/patient anonymity. Requests to access the datasets should be directed to the corresponding author.

## Ethics Statement

The studies involving human participants were reviewed and approved by Clinical Research Ethics Committee of The First Affiliated Hospital, College of Medicine, Zhejiang University. Written informed consent to participate in this study was provided by the participants' legal guardian/next of kin. Written informed consent was obtained from the individual(s), and minor(s)' legal guardian/next of kin, for the publication of any potentially identifiable images or data included in this article.

## Author Contributions

YZ and QC wrote the manuscript together. HLi provided the clinical data. YZ, QC, HLu, and YQ participated in the experiment. QY and CW contributed to conception and design of the study. All authors contributed to manuscript revision, read, and approved the submitted version.

## Conflict of Interest

The authors declare that the research was conducted in the absence of any commercial or financial relationships that could be construed as a potential conflict of interest.

## Publisher's Note

All claims expressed in this article are solely those of the authors and do not necessarily represent those of their affiliated organizations, or those of the publisher, the editors and the reviewers. Any product that may be evaluated in this article, or claim that may be made by its manufacturer, is not guaranteed or endorsed by the publisher.

## Supplementary Material

Supplementary Figure S1 Genetic diagnosis. **(A)** A schematic showing the filtering procedure of variants obtained by whole-exome sequencing. The number indicates the number of variants passed for each step. **(B)** Pathogenic assay for FGD1^N424D^ variant.

Supplementary Table S1 The 11 corresponding SNPs. OMIM, Online Mendelian Inheritance in Man; ACMG, American College of Medical Genetics and Genomics.
