# Supplementary figures and images for: FGD1 Variant Associated With Aarskog–Scott Syndrome
Source: Front Pediatr. 2022 Jul 14;10:888923. doi: 10.3389/fped.2022.888923 (PMC9329920; doi:10.3389/fped.2022.888923)

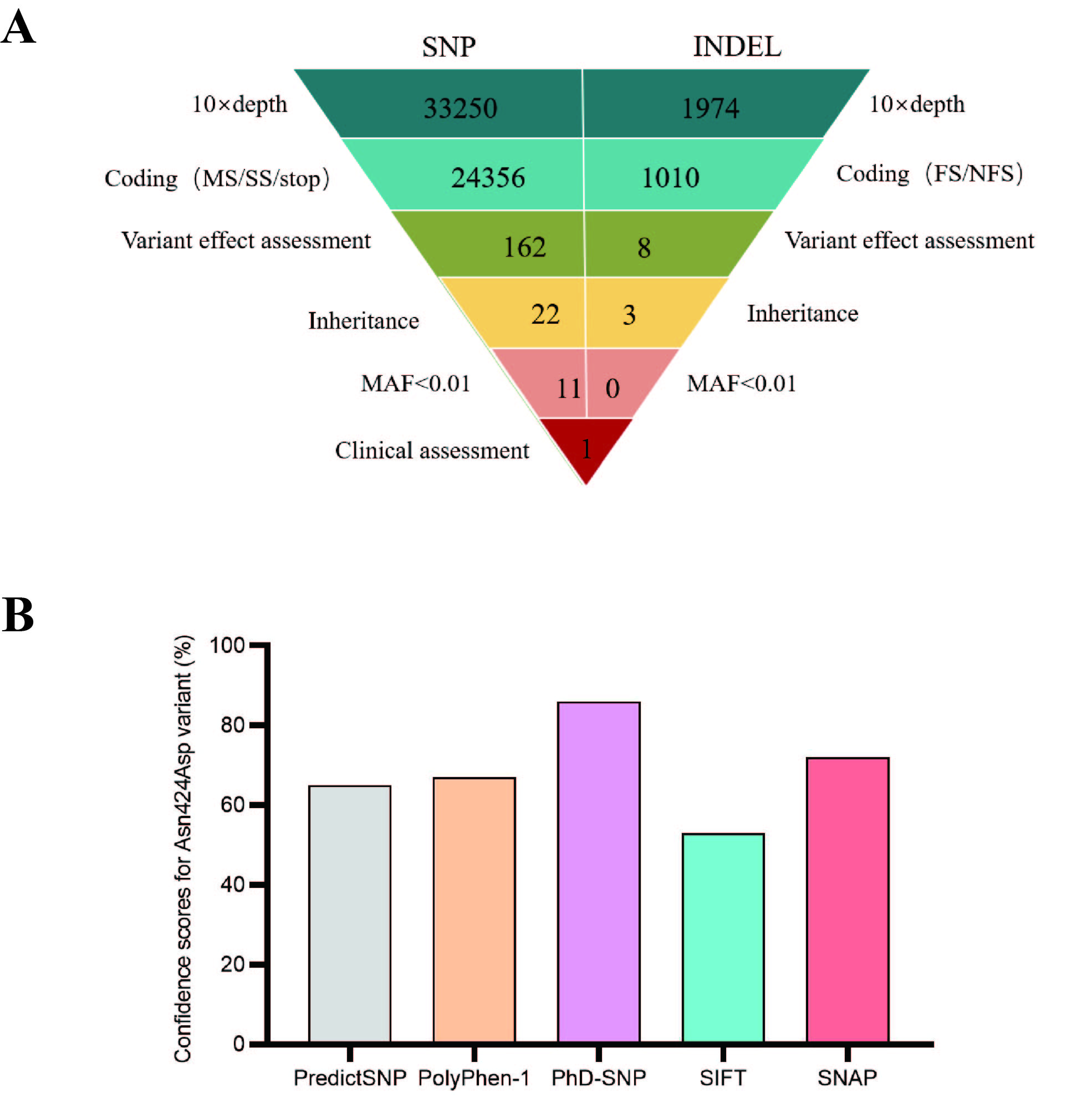

Supplement: Supplementary Figure S1 — Genetic diagnosis. (A) A schematic showing the filtering procedure of variants obtained by whole-exome sequencing. The number indicates the number of variants passed for each step. (B) Pathogenic assay for FGD1N424D variant. [file Figure_1.jpg]
